# Functional Neural Correlates of Attentional Deficits in Amnestic Mild Cognitive Impairment

**DOI:** 10.1371/journal.pone.0054035

**Published:** 2013-01-11

**Authors:** Nicholas T. Van Dam, Mary Sano, Effie M. Mitsis, Hillel T. Grossman, Xiaosi Gu, Yunsoo Park, Patrick R. Hof, Jin Fan

**Affiliations:** 1 Department of Psychology, Queens College, City University of New York, Flushing, New York, United States of America; 2 Department of Psychiatry, Mount Sinai School of Medicine, New York, New York, United States of America; 3 Department of Neuroscience, Mount Sinai School of Medicine, New York, New York, United States of America; 4 Department of Neurology, Mount Sinai School of Medicine, New York, New York, United States of America; 5 Alzheimer’s Disease Research Center, Mount Sinai School of Medicine, New York, New York, United States of America; 6 Mount Sinai Memory and Aging Center, Mount Sinai School of Medicine, New York, New York, United States of America; 7 Friedman Brain Institute, Mount Sinai School of Medicine, New York, New York, United States of America; Nathan Kline Institute and New York University School of Medicine, United States of America

## Abstract

Although amnestic mild cognitive impairment (aMCI; often considered a prodromal phase of Alzheimer’s disease, AD) is most recognized by its implications for decline in memory function, research suggests that deficits in attention are present early in aMCI and may be predictive of progression to AD. The present study used functional magnetic resonance imaging to examine differences in the brain during the attention network test between 8 individuals with aMCI and 8 neurologically healthy, demographically matched controls. While there were no significant behavioral differences between groups for the alerting and orienting functions, patients with aMCI showed more activity in neural regions typically associated with the networks subserving these functions (e.g., temporoparietal junction and posterior parietal regions, respectively). More importantly, there were both behavioral (i.e., greater conflict effect) and corresponding neural deficits in executive control (e.g., less activation in the prefrontal and anterior cingulate cortices). Although based on a small number of patients, our findings suggest that deficits of attention, especially the executive control of attention, may significantly contribute to the behavioral and cognitive deficits of aMCI.

## Introduction

Alzheimer’s disease (AD) first presents as mild cognitive impairment (MCI) in terms of memory loss or decline in other cognitive functions (e.g., attention). Studies suggest that the conversion rate of MCI to AD is 41% over a 1-year period and 64% over a 2-year period [1]. Amnestic MCI (aMCI) has such a high conversion rate to AD that it is considered by some as a prodromal phase of AD [2], [3]. While the economic burden attributable to MCI is quite small [2], the annual cost of patient care in AD is more than $100 billion in the United States alone [4]. Global projections suggest that delaying the progression and onset of AD by as little as one year could have a massive impact on the global economic burden of the disease [5]. Although AD is primarily characterized by memory impairments [6], there is accumulating evidence that attentional deficits occur during relatively early stages of the disease [7]–[11]. In fact, some research has shown that efficiency of attentional processes discriminate between patients with mild AD and the healthy elderly [12]. Further, other studies have shown that attentional impairment is a predictor of cognitive decline in early stages of probable AD [13]. Thus alterations in attentional function may be a useful diagnostic marker, prognostic indicator, and potential point of intervention, among those with prodromal AD.

Attention refers to the activity of a set of brain networks that can influence the priority of the computations of other brain networks for access to consciousness [14]. Impairments of attention may contribute to functional decline in other cognitive domains, such as memory in aging and dementia [15]. Although deficits in attention [16] and executive control of attention [17] are usually the initial deficits observed following emergence of amnestic symptoms during early stages of AD [17], [18], little is known about the pathophysiological basis of these deficits relative to memory impairments. Behavioral studies of attention mechanisms, in combination with new technologies such as functional neuroimaging, may assist in better identifying the pathophysiology of deficits associated with AD [19], as well as its precursor, aMCI [3].

One attentional network theory has conceptualized attention as comprised of three functionally and anatomically defined brain networks of alerting, orienting, and executive control [20]–[22]. The alerting network involves tonically maintaining the alert state and phasically responding to a warning signal. It involves the thalamic, frontal, and parietal regions, and temporoparietal junction [23]. The orienting network subserves the functions of endogenous and exogenous selecting of information from among numerous sensory inputs. The key neural substrates for the orienting network include the superior parietal lobule and frontal eye fields [23]. The executive control function of attention involves the engagement of more complex mental operations during monitoring and resolving conflict between computations. The anterior cingulate cortex (ACC) and dorsolateral prefrontal cortex (DLPFC) are involved in this network [23]. This attention network theory [20]–[22] can be mapped onto the stimulus-driven and goal-directed model of Corbetta and Shulman [24] by considering the (re)orienting function as the hub of top-down and bottom-up convergence [25]. In this way, the phasic alerting network can be perceived as a potential bottom-up influence, while the executive control network can be perceived as a potential top-down influence on selective attention.

Previous findings have suggested that attention deficits contribute to the symptomatic profile of AD. Deficits have been documented in the alerting and orienting networks [12], [15], [17], [26]–[35], as well as in the executive control of attention among individuals with AD [9], [11], [12], [17], [36]–[42]. Further evidence has shown broad deficits of general executive function in AD [17], [43]–[49]. A behavioral study using the attention network test (ANT) showed selective impairments in executive control and an interaction between orienting and executive control in AD [10]. These various attention deficits, observed in AD, have been previously explained as a disruption of the basal forebrain cholinergic system and cortico-cortical tracts connecting distinct cortical regions [17], [18]. Nonetheless, the neural basis of attention deficits in AD is still not fully understood [17]. One structure of potential interest is the ACC. Converging evidence has indicated that the ACC plays a key role in the network subserving executive control of attention [50], [51]. In AD, several studies have shown deficits of the ACC [52]–[58]. These findings suggest that abnormalities in this structure may underlie deficits in executive control of attention [59]. Deficits of executive control of attention in AD (and its precursor aMCI), implicating neural areas such as the ACC, would fill gaps in the existing literature.

In the present study, we assessed the three attentional functions of alerting, orienting, and executive control, and the corresponding neural networks in patients with aMCI. Participants completed the ANT, which we previously developed and have validated in both healthy controls and psychiatric patients [20], [21], [23], [60], [61], while undergoing functional magnetic resonance imaging (fMRI). We predicted that, compared to healthy age-matched controls, patients with aMCI might show deficits in alerting and orienting, but more likely, less efficient executive control associated with a greater conflict effect and reduced ACC (and other prefrontal cortical) activation.

## Materials and Methods

### Participants

We recruited 19 individuals with aMCI and 15 healthy controls (HC) via the Alzheimer’s Disease Research Center (ADRC) at Mount Sinai School of Medicine (MSSM). This study was approved by the MSSM institutional review board (IRB) and signed consent forms were collected from the participants. While MCI participants are not typically without capacity as they are not demented, standard MSSM consent procedures in this cohort requires that each participant be given adequate time to ask questions about the study so that they are fully informed with regard to study procedures and participants must demonstrate understanding of procedures by paraphrasing key aspects of the study. If a subject appears to lack understanding, the legally authorized representative provides consent as per MSSM IRB guidelines.

Individuals were assessed and diagnosed through the Clinical Core of the ADRC using the National Alzheimer Coordinating Center’s Uniform Data Sets (UDS). The evaluation includes a semi-structured interview of the participant and an informant regarding clinical symptoms and chronology, as well as medical, neurological and neuropsychiatric examination, and neuropsychological testing. Amnestic MCI was diagnosed according to previously used, and established criteria [62], [63], in the present study this included (but was not limited to) a Mini-Mental State Exam (MMSE [64]) score of 24 or higher, performance on delayed recall of the first paragraph of the Wechsler Memory Scale [65] using age and education adjusted scores, and no significant impairment in social or occupational function. HCs underwent the same evaluations, with Wechsler Memory Scale performance falling within the normal range for age and education. The evaluation also included administration of the Clinical Dementia Rating scale (CDR: [66]). Amnestic MCI patients had a CDR of 0.5 while healthy controls predominantly had a CDR of 0. HCs were not excluded for a CDR = 0.5, since those with ‘mild’ dementia are not necessarily representative of individuals who are likely to progress to AD (as are those with aMCI), and some minimal dementia might be anticipated in a normal geriatric sample. Determination of aMCI or normal control status was accomplished via clinical consensus following complete review by the evaluating physician and an ADRC neuropsychologist. Of the 34 originally recruited individuals, 10 MCIs and 4 HCs could not undergo MRI scans for numerous reasons (e.g., arthritis prevented comfortable position on scanner bed, extreme difficulty seeing the visual display, or metallic implant). Another MCI and 2 HCs were excluded due to excessive head motion (>3 mm within a run). An additional HC was excluded due to reaction time (RT) and accuracy that had an absolute distance from the mean of more than 2 standard deviations (SD). Our final sample size was 8 MCIs and 8 HCs. All participants were right-handed and had normal or corrected-to-normal vision. Corrective lenses were used as necessary and visual acuity was tested in advance to ensure participants could view the arrows clearly. Demographic and diagnostic information is provided in Table 1.

**Table 1:** Demographic characteristics and statistical comparisons.

	HC (n = 8)	aMCI (n = 8)	
	M (SD)	M (SD)	p
**Age**	74.6 (9.2)	77.6 (7.0)	0.48
**Education**	16.9 (2.4)	14.6 (3.2)	0.12
**MMSE**	28.8 (1.4)	27.1 (1.8)	0.06
**CDR**	0.13 (0.23)	0.50 (0.00)	0
	**%**	**%**	**p**
**Male**	25	50	0.6
**Race**			0.3
White	100	62.5	
African American	0	12.5	
Asian	0	12.5	
No Answer	0	12.5	

HC = Healthy control; aMCI = amnestic Mild Cognitive Impairment; MMSE = Mini-Mental State Exam; CDR = Clinical Dementia Rating; *p* = *p* value resulting from statistical test.

### Task and Procedure

#### Attention Network Test

The ANT [14], [20] was re-designed for the present study to optimize attentional contrasts in an elderly population. The ANT, modified for use in a geriatric population (ANT-G) used three cue conditions (no cue, double cue, and spatial cue) with two target conditions (congruent and incongruent). In this version, the cue-to-target interval was held constant at 400 ms and there were no invalid cues (cues were always valid indicators of target location). As with previous versions of the ANT, a central arrowhead points leftward or rightward and there are two arrowheads on either side of the central arrowhead. All four flanking arrowheads can face the same or opposite direction as the central arrowhead, which is the target. The target and flanker were presented for 2500 ms, though the response window was open for an additional 1500 ms, followed by a 2500 ms inter-trial interval.

**Figure 1:**
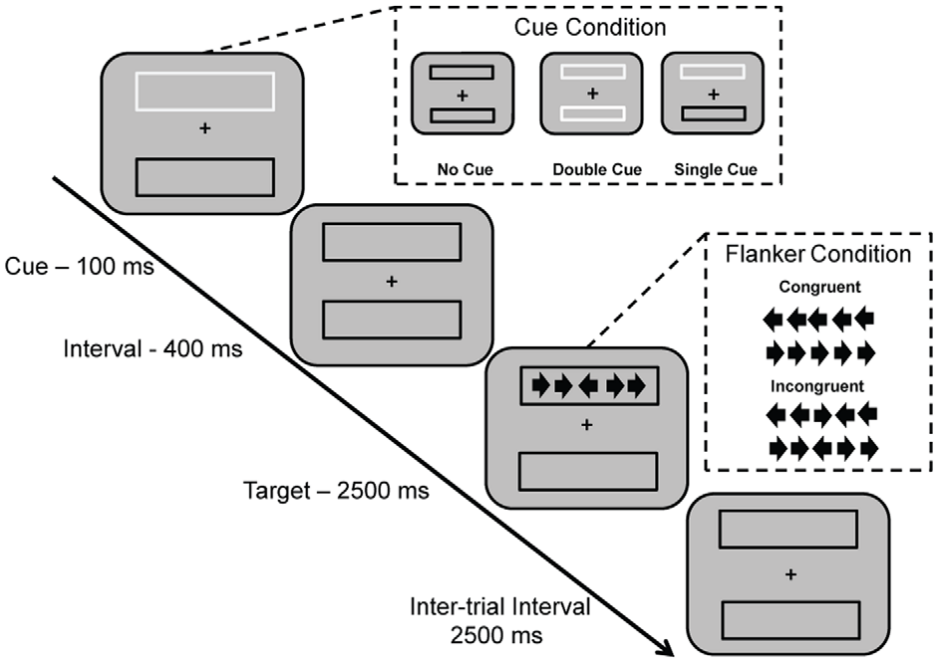
Schematic of modified Attention Network Test for geriatric samples (ANT-G). In each trial, depending on the cue condition (no cue, double cue, spatial cue), a box changes from black to white (flashes) for 100 ms. After 400 ms, the target (center arrow) and four flanker arrows (two on either side of center arrow, congruent or incongruent with center arrow) are presented for 2500 ms. The participant makes a response to indicate the direction of the center arrow (left or right). The response window remains open for an additional 1500 ms after the termination of the target (4000 ms in total for the response window), proceeding into the 2500 ms inter-trial interval.

In the ANT-G, the shape of arrowheads was revised to enlarge the vertical dimension (4× the original dimension). Additionally, the visual angle for orienting (up/down) was enlarged by 30% compared to the original version [20] to make target detection easier for elderly participants. The participants’ task was to identify the direction of the center arrow by pressing a button with their left index finger if the target was pointing to the left and a button with the right index finger for the right direction if the target was pointing right. In the ANT-G, participants completed 3 blocks of 32 trials, for a total of 96 trials. In each block, 16 blank periods (no cue and no target presented) of equivalent length each to a single trial, were used to jitter the presentation of trials. Details of this version of the ANT-G are illustrated in Figure 1.

Each of the three attentional networks is operationally defined as a comparison of the performance (RT and error rate) of one condition and the appropriate reference condition, increasing the likely of a positive score for each attentional network. For the alerting network, the effect is defined as *RT_no_*_ cue_ – *RT_double cue._* For the orienting network, the effect is defined as *RT_double cue_ – RT_single cue_*. For the executive control network, the conflict effect is defined as *RT_flanker incongruent_ – RT_flanker congruent_.* Performance in error rate was computed using the exact same formulae. Error rates were computed as number of incorrect trials for a given trial type (condition) divided by total number of trials presented for that same trial type.

Prior to implementation in the scanner, participants completed a training session of the ANT-G with step-by-step instructions for 6 trials, followed by a practice block containing 24 trials. This was done on a PC outside the scanner. After participants completed this training session, they then completed 32 trials of the ANT-G in an MRI simulator (Psychology Software Tools, Inc., Pittsburgh, PA), which provided a realistic approximation of the MRI scanner, including simulation of the noises related to the scan sequences, to permit acclimatization to the scanner environment.

#### fMRI data acquisition and analysis

All MRI data were obtained using a 3 T Siemens Allegra MRI system at MSSM. Foam padding was used to minimize subject head movements. All images were acquired along axial planes parallel to the anterior commissure-posterior commissure line. A high-resolution T2-weighted anatomical volume of the whole brain was acquired with a turbo spin-echo pulse sequence. The fMRI imaging was performed using a gradient-echo echo-planar imaging (GE-EPI) sequence with the following protocol: 40 axial slices, 4 mm-thick, and skip = 0 mm, TR = 2500 ms, TE = 27 ms, flip angle = 82°, FOV = 240 mm, and matrix size = 64×64. Slices were obtained corresponding to the T2-weighted anatomical images. Three series of EPIs corresponding to the three runs were acquired. Each series started with 2 dummy volumes before the onset of the task to allow for equilibration of T1 saturation effects, followed by 165 image volumes. Each of the 3 runs of the ANT-G was preceded and followed by a 30-s fixation period.

Event-related analyses of the fMRI data from the tasks were conducted using the statistical parametric mapping package (SPM8; Wellcome Trust Centre for Neuroimaging, London, UK). Functional scans were adjusted for slice timing, realigned to the first volume, co-registered to the T2 image, normalized to a standard template (MNI, Montreal Neurological Institute), resampled to 2×2×2 mm voxel size, and spatially smoothed with an 8×8×8 mm full-width-at-half-maximum Gaussian kernel. General linear modeling [67] was then conducted for the functional scans from each participant by modeling the observed event-related blood oxygenation level-dependent (BOLD) signals and regressors to identify the relationship between the task event and the BOLD signal. Regressors were created by convolving a train of delta functions representing the sequence of onsets of cues and targets with the default SPM basis function, which consists of a synthetic hemodynamic response function composed of two gamma functions [68].

Regressors were generated for each of the three cue conditions (3 regressors: double cue, single/spatial cue, no cue; all cue locked), as well as their interactions with the congruent and incongruent flanker conditions (6 regressors; all target locked), for a total of 9 regressors. Six parameters generated during motion correction were entered as covariates. The *alerting* effect was examined by computing the double cue minus no cue contrast, for these cue-locked regressors. The *orienting* effect was examined by computing the single cue minus double cue contrast, for these cue-locked regressors. The executive control or *flanker conflict* effect was examined by computing all incongruent minus all congruent conditions for the six target-locked regressors.

Contrast images from all participants were entered into a second-level group analysis conducted with a random-effect model. The group differences represent the “activation” differences rather than the baseline differences. This is consistent with the ANT score computation because the attentional network test is based on cognitive subtraction. Significant activations of interest were identified with voxel-wise *p*<0.05 in conjunction with an extent threshold of k = 120 (*t* ≥1.89 for single subject contrasts and *t* ≥1.76 for group contrasts, resampled voxel size). This threshold was determined using a Monte Carlo simulation that modeled the entire imaging volume iteratively, using an individual voxel type I error rate of *p*<.05 and 8 mm FWHM smoothing. A cluster extent threshold was determined across 1,000 iterations to set the overall type I error rate to.05 (i.e., p*<.*05), given the parameters of data acquisition [69].

## Results

### Group Demographics

Table 1 shows that the aMCI and HC groups did not significantly differ on age (*t*_(14)_ = 0.73, *p* = .48, *d* = 0.39), education (*t*_(14)_ = 1.60, *p* = 0.13, *d* = 0.87), gender, (*χ^2^*_(1)_ = 1.07, *p* = 0.30), or race (*χ^2^*_(3)_ = 3.69, *p* = 0.30). Table 1 also shows that the groups differ, at a level near but not reaching significance, on Mini-Mental State Exam [64] scores, at the time of evaluation (*t*_(14)_ = 2.02, *p* = 0.06, *d* = 1.13). Scores ranged from 24 to 30. The groups also differ on the CDR [66], with 100% of individuals in the aMCI group exhibiting scores of 0.5 (very mild dementia) and 25% (2 individuals) exhibiting scores of 0.5 in the HC group, *t*_(14)_ = 4.58, *p*<0.001, *d* = 2.43. Scores did not exceed 0.5.

### Behavioral Results

On average, the median RT was 44.4 ms less than the mean RT. Only 2 of 16 individuals showed higher median than mean RTs. Along with an average *SD* of 276.43 ms, the findings suggested positive skew. Thus, we opted to use median reaction time as the basis for our analyses. Because there were an equal number of trials in each of the experimental conditions, and equal sample sizes in both groups, we were not concerned about bias in median reaction times [70].

**Figure 2:**
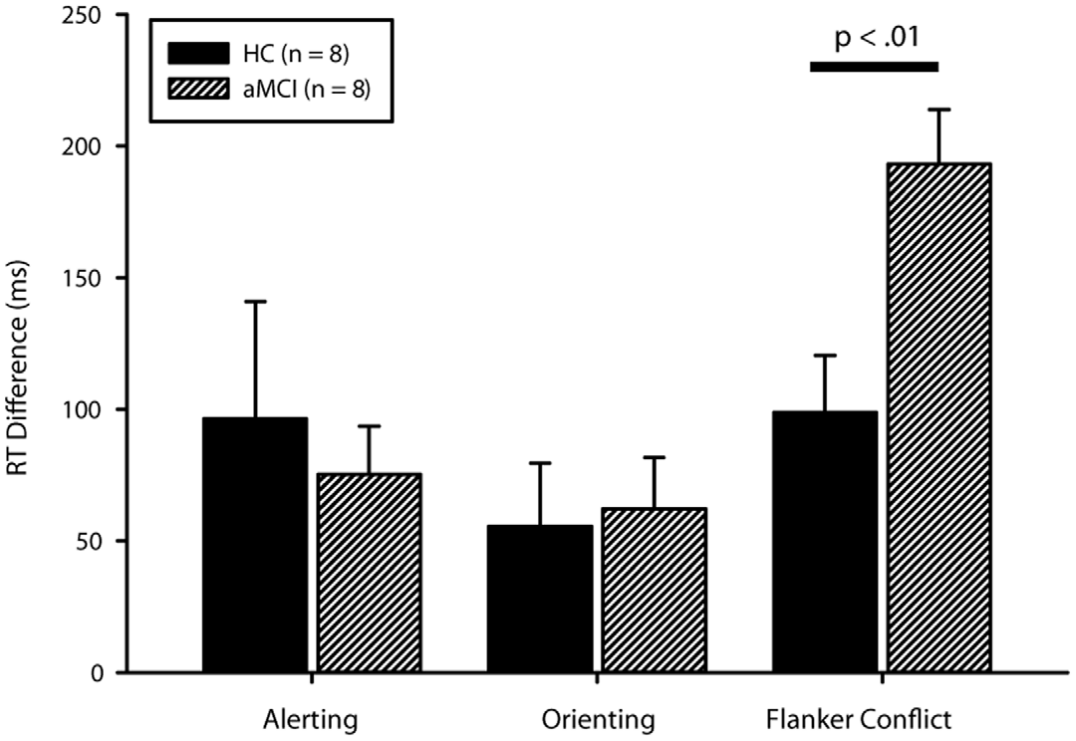
Group differences in median reaction time by attentional function. Only the group difference for the flanker conflict (executive control) effect reached significance at *p*<0.05 (actual *p*<0.01). Error bars represent standard error.

Group differences in the accuracy of alerting approached significance (*t*_(14)_ = 1.97, *p* = 0.07), while orienting (*t*_(14)_ = 0.62, *p* = 0.55) and executive control (*t*_(14)_ = 0.10, *p* = 0.92) did not differ statistically (see Table 2). It is important to note that while the alerting effect on error rate approached significance, there was no statistical difference in error rate on any of the individual trial types or overall performance (see Table 2). This statistical equivalence in terms of accuracy is important because it indicates a comparable number of correct trials to be modeled for the neuroimaging analysis. This lack of difference in accuracy also led us to retain error trials for the imaging contrasts. There were no significant differences between groups in the reaction times of the alerting (*t*_(14)_ = 0.44, *p* = 0.67) or orienting functions (*t*_(14)_ = 0.21, *p* = 0.84), though there was a large significant difference in the executive function, *t*_(14)_ = 3.16, *p* = 0.007, Cohen’s *d* = 1.7 (see Table 3, Figure 2).

**Table 2:** Error rates between groups for trial conditions and attentional effects.

	HC (n = 8)	aMCI (n = 8)	Cohen’s d
**Cue Condition**			
None	6.00 (11.96)	8.00 (8.32)	0.21
Double	10.00 (15.78)	6.00 (7.05)	0.35
Single/Spatial	9.00 (13.17)	6.00 (7.09)	0.3
**Flanker Condition**			
Congruent	6.00 (8.43)	5.00 (3.94)	0.16
Incongruent	11.00 (19.08)	9.00 (10.34)	0.14
**Effect**			
Alerting	−3.88 (4.22)	1.38 (6.26)	1.05#
Orienting	1.50 (3.33)	0.00 (6.00)	0.33
Executive Control	4.62 (11.26)	4.12 (7.64)	0.06
**Overall**	5.00 (6.48)	7.00 (7.11)	0.31

# *p* = 0.07.

**Table 3:** Reaction time between groups for trial conditions and attentional effects.

	HC (n = 8)		aMCI (n = 8)	Cohen’s d
**Cue Condition**				
None	1123.13 (210.72)		1096.75 (178.98)	0.14
Double	1026.75 (148.52)		1021.38 (194.66)	0.03
Single/Spatial	971.19 (149.60)		959.19 (202.62)	0.07
**Flanker Condition**				
Congruent	989.06 (188.36)		926.88 (172.67)	0.37
Incongruent	1088.06 (149.48)		1120.06 (203.13)	0.19
**Effect**				
Alerting	96.38 (126.48)		75.38 (51.31)	0.23
Orienting	55.56 (67.54)		62.19 (55.49)	0.11
Executive Control	99.00 (60.78)		193.19 (58.40)	1.69**
**Overall RT**	1049.38 (166.62)		1020.25 (185.74)	0.18

** *p*<0.01.

Note: Reaction time (RT) analyses were performed using Median RT due to skew.

### Functional Neuroimaging Results

**Figure 3:**
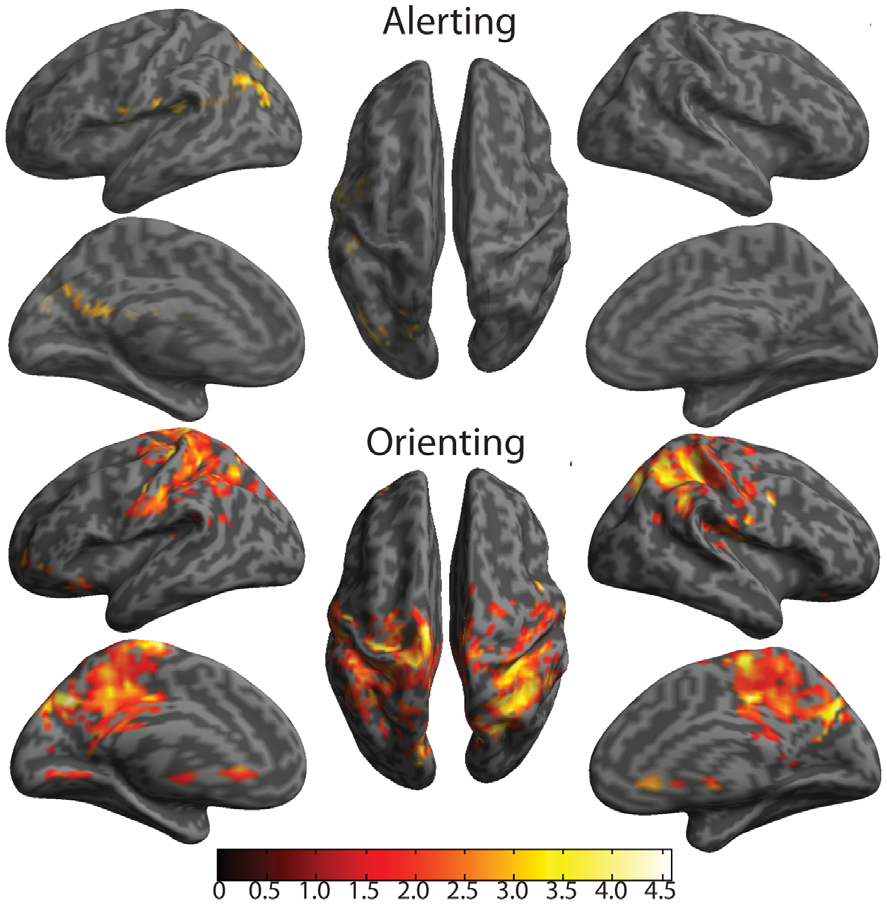
Cortical surface maps of Alerting and Orienting effects for aMCI>HC contrast. All represented activity has been thresholded at *p*<0.05 for height and k = 120 (p<0.05) for cluster extent to set the nominal alpha level to *p*<0.05 for multiple comparisons (corresponds to *t t* ≥1.76), based on Monte Carlo simulation of our data.

Due to comparable performances between groups in the components of the *alerting* condition, we conducted analyses examining potentially greater (compensatory) activity in the aMCIs vs. HC. Differences in BOLD activity, related to the alerting effect, were present despite no behavioral differences (see Table 3, Figure 3). Notably, aMCIs showed greater activation of the temporoparietal junction (TPJ; *x* = −48, *y* = −36, *z* = 20), precuneus (*x* = −2, *y* = −48, *z* = 18), and angular gyrus (*x* = −48, *y* = −68, *z* = 34), all in the left hemisphere. Because posterior cingulate cortex (PCC), precuneus, and angular gyrus are prominent nodes of the default mode network (DMN) [71], the difference between aMCI and HC may indicate less deactivation in the aMCI. Greater TPJ activation and left laterality is consistent with our previous findings using the original ANT [23].

Similar to the *alerting* effect, and due to comparable performances between groups in the *orienting* condition, we conducted analyses examining potentially greater (compensatory) activity in the aMCIs vs. HC. Differences in BOLD activity, related to the orienting effect, were also present despite no behavioral differences (see Table 4, Figure 3). MCIs predominantly showed greater activation in areas traditionally associated with the orienting function (i.e., posterior parietal regions [23]). Areas of greater activity in aMCIs included the superior parietal lobule and pre- and postcentral gyri, and PCC, all regions bilaterally. For the PCC, the greater activation in aMCI might be related to less deactivation in this brain region.

**Table 4:** Greater network-related activation in aMCI compared to HC.

Region	L/R	BA	MNI coordinates			Z	p	k
			x	y	z			
**Alerting**								
Superior temporal gyrus	L	41	−48	−36	20	3.16	0.001	191
Postcentral gyrus	L	43	−52	−18	18	2.39	0.008	
Angular gyrus	L	39	−48	−68	34	2.54	0.005	335
Middle occipital lobe	L	19	−28	−78	42	2.54	0.006	
Middle occipital lobe	L	39	−38	−80	28	2.52	0.006	
Superior parietal lobule	L	7	−26	−72	50	2.2	0.014	
Superior parietal lobule	L	7	−16	−76	50	2.1	0.018	
Middle occipital lobe	L	39	−36	−70	22	2.06	0.02	
Precuneus	L	30	−2	−48	18	2.32	0.01	227
Cuneus	L	31	−8	−64	28	2.02	0.022	
Calcarine	R	17	6	−66	18	1.68	0.046	
Superior temporal lobe	L	22	−58	−8	6	2.3	0.011	125
Insula	L	13	−42	0	12	2.01	0.022	
Cerebellum 4/5	R	30	14	−42	−16	2.11	0.018	160
Cerebellum 6	R	37	26	−50	−30	2.01	0.022	
Cerebellum 1	R		36	−58	−30	1.99	0.023	
**Orienting**								
Paracentral lobule	L	6	−4	−18	72	3.9	0	19649
Supplementary motor area	R	4	8	−20	62	3.89	0	
Precuneus	L	7	−8	−74	38	3.64	0	
Cuneus	R	7	14	−68	34	3.54	0	
Superior parietal lobule	L	7	−18	−40	42	3.53	0	
Inferior parietal lobule	R	7	28	−52	56	3.47	0	
Precentral gyrus	R	6	44	−2	36	3.41	0	
Precuneus	R	19	18	−68	42	3.3	0	
Postcentral gyrus	R	3	32	−38	56	3.22	0.001	
Precuneus	L	3	−14	−38	72	3.2	0.001	
Postcentral gyrus	L	3	−34	−30	52	3.18	0.001	
Paracentral lobule	L	4	−6	−26	66	3.17	0.001	
Inferior parietal lobule	L	40	−28	−48	40	3.15	0.001	
Superior parietal lobule	L	7	−30	−64	46	3.11	0.001	
Posterior cingulate gyrus	L	31	0	−44	46	3.08	0.001	
Angular gyrus	R	40	44	−44	36	3.07	0.001	
Cuneus	L	19	−12	−84	32	2.96	0.002	
Supramarginal gyrus	R	40	44	−36	42	2.87	0.002	
Precentral gyrus	L	6	−30	−22	62	2.7	0.003	
Superior parietal lobule	R	7	14	−68	56	2.63	0.004	
Rolandic operculum	R	43	40	−14	20	2.61	0.005	
Lingual gyrus	L	18	−8	−58	4	2.5	0.006	
Posterior cingulate gyrus	R	31	8	−38	42	2.42	0.008	
Supramarginal gyrus	L	48	−48	−26	28	2.41	0.008	
Lingual gyrus	L	18	−4	−68	4	2.38	0.009	

**Figure 4:**
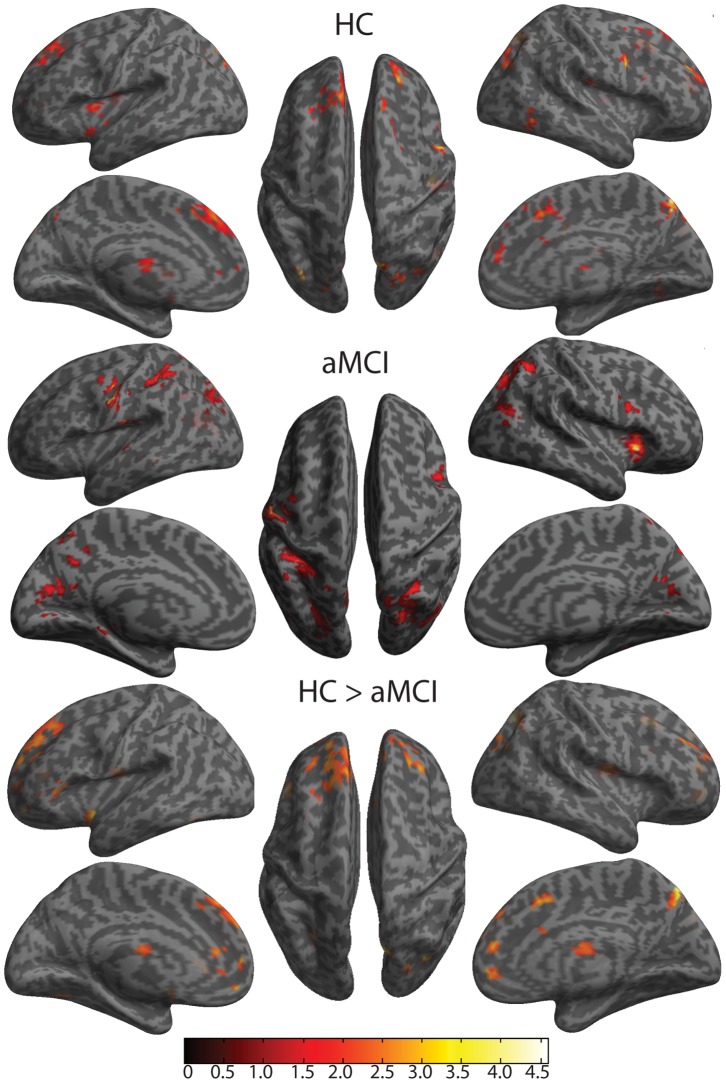
Cortical surface maps of Executive Control effect for HC, aMCI, and group contrast (HC>aMCI). The top set is the contrast between flanker incongruent and flanker congruent conditions in Healthy Controls (HC). The middle set is the contrast between flanker incongruent and flanker congruent conditions in patients with amnestic Mild Cognitive Impairment (aMCI). The bottom set is the contrast between HC and aMCI. All represented activity has been thresholded at *p*<0.05 for height and k = 120 (p<0.05) for cluster extent to set the nominal alpha level to *p*<0.05 for multiple comparisons, based on Monte Carlo simulation of the data.

Consistent with the primary hypothesis, there were significant behavioral differences in relation to the flanker conflict effect. Behaviorally, the HC group showed a significantly smaller difference between the congruent and incongruent conditions than the aMCI group (see Figure 2). The aMCI group exhibited corresponding BOLD differences, such that there was less activation in the medial prefrontal regions, especially prefrontal cortex (Brodmann area 10) and ACC, which also extended to the DLPFC (see Table 5, Figure 4). Differences are consistent with previous findings for the flanker conflict effect [23] and with recent discussion about a dual architecture for cognitive control [72].

**Table 5:** BOLD activation related to executive control in HC, aMCI, and HC>aMCI.

Region	L/R	BA	MNI coordinates			Z	p	k
			x	y	z			
**HC**								
Precuneus	R	7	10	−72	54	3.91	0	678
Superior occipital gyrus	R	7	26	−76	42	2.68	0.004	
Cuneus	R	19	20	−82	42	2.67	0.004	
Precuneus	L	7	−4	−80	44	2.67	0.004	
Superior occipital gyrus	R	19	26	−74	26	2.38	0.009	
Middle occipital gyrus	R	19	34	−80	38	2.35	0.009	
Rolandic operculum	R	43	42	−16	16	3.73	0	135
Middle occipital gyrus	L	19	−28	−82	38	3.14	0.001	126
Precentral gyrus	R	44	54	10	32	3.1	0.001	206
Thalamus	L		−12	−6	12	3.01	0.001	523
Thalamus	R		6	−10	6	2.3	0.011	
Anterior cingulate gyrus	R	32	2	50	12	2.79	0.003	232
Insula	L	13	−36	2	6	2.76	0.003	312
Putamen	L		−30	8	−2	2.39	0.008	
Supplementary motor area	L	32	0	16	48	2.73	0.003	1614
Superior frontal gyrus, medial	L	32	−8	36	44	2.65	0.004	
Middle frontal gyrus	L	8	−24	26	42	2.29	0.011	
Anterior cingulate gyrus	R	24	2	22	32	2.1	0.018	
Middle frontal gyrus	L	9	−26	38	34	1.96	0.025	
Superior frontal gyrus	R	46	24	44	24	2.69	0.004	194
Superior frontal gyrus	R	10	22	56	16	2.3	0.011	
Middle frontal gyrus	R	46	24	56	24	2.19	0.014	
Inferior temporal gyrus	R	37	50	−58	−4	2.46	0.007	140
Superior frontal gyrus	R	32	16	26	50	2.38	0.009	121
Middle frontal gyrus	R	8	24	12	52	2.12	0.017	
Superior frontal gyrus	R	6	28	2	56	1.91	0.028	
**aMCI**								
Insula/Inferior frontal gyrus	R	47	32	18	0	4.26	0	390
Insula	R	13	32	20	10	3.24	0.001	
Postcentral gyrus	L	3	−52	−14	32	4.03	0	376
Postcentral gyrus	L	3	−42	−12	38	3.45	0	
Superior occipital gyrus	L	23	−20	−64	26	3.72	0	2255
Superior occipital gyrus	R	7	26	−74	42	3.57	0	
Calcarine sulcus	L	17	−4	−66	12	3.54	0	
Superior occipital gyrus	R	19	26	−64	24	3.26	0.001	
Superior occipital gyrus	L	19	−24	−82	36	3.11	0.001	
Middle occipital gyrus	R	39	40	−72	22	2.95	0.002	
Calcarine sulcus	R	17	4	−66	14	2.87	0.002	
Precuneus	R	5	12	−60	60	2.58	0.005	
Middle temporal gyrus	R	39	46	−66	16	2.42	0.008	
Superior parietal lobule	L	7	−22	−72	46	2.05	0.02	
Inferior frontal gyrus	R	44	44	8	26	3.56	0	222
Superior parietal lobule	L	7	−30	−48	70	3.5	0	1037
Precuneus	L		−14	−58	36	3.38	0	
Inferior parietal lobule	L	40	−30	−44	40	3.24	0.001	
Superior parietal lobule	L	7	−26	−50	50	2.94	0.002	
Inferior parietal lobule	L	40	−38	−44	54	2.77	0.003	
Superior parietal lobule	L	7	−26	−56	68	2.34	0.01	
Superior temporal lobe	L	22	−60	−10	8	3.29	0	238
Superior temporal lobe	L	22	−60	−18	10	3.26	0.001	
Fusiform gyrus	R	37	42	−46	−22	2.47	0.007	135
Inferior temporal gyrus	R	37	44	−44	−12	2.32	0.01	
Parahippocampal gyrus	L	37	−22	−34	−8	2.23	0.013	172
Vermis 3			2	−36	−4	2.06	0.02	
Cerebellum 4/5	L	30	−8	−42	−12	1.99	0.023	
**HC>aMCI**								
Precuneus	R	7	8	−74	52	3.28	0.001	224
Anterior cingulate cortex	L	32	0	50	4	3.21	0.001	2788
Middle frontal gyrus (medial)	L	10	−8	50	−6	2.99	0.001	
Superior frontal gyrus (medial)	L	8	−4	40	52	2.87	0.002	
Anterior cingulate Gyrus	R	32	6	16	44	2.64	0.004	
Middle frontal gyrus	R	46	26	58	24	2.58	0.005	
Superior frontal gyrus	L	46	−26	54	22	2.46	0.007	
Middle frontal gyrus	L	9	−24	42	34	2.3	0.011	
Middle frontal gyrus	L	46	−30	40	26	2.23	0.013	
Superior frontal gyrus	L	9	−22	30	44	2.2	0.014	
Superior frontal gyrus	R	10	14	58	24	1.99	0.023	
Anterior cingulate gyrus	L	32	−2	34	30	1.92	0.028	
Middle occipital lobe	R	19	32	−80	28	2.98	0.001	121
Insula	L	13	−34	0	−12	2.73	0.003	124
Thalamus	L		−12	−12	16	2.52	0.006	346
Thalamus	R		8	−8	12	2.39	0.008	
Inferior frontal gyrus	L	47	−46	22	0	2.39	0.008	125
Middle frontal gyrus	R	46	28	40	26	2.38	0.009	141
Cerebellum 6	L	19	−32	−62	−20	2.34	0.01	130
Fusiform gyrus	L	37	−26	−58	−14	1.98	0.024	

## Discussion

In the present investigation of a modified version of the attention network test (ANT-G) in healthy controls and individuals with aMCI, there were notable attention deficits among patients with aMCI. While the groups exhibited no significant behavioral differences in the alerting or orienting networks, consistent with some prior work (e.g., [10]), there were significant neural differences for these networks. Since performance was equivalent across groups, but the aMCI group exhibited increased neural activation in the alerting and orienting networks, one might argue that compensatory activity contributed to behavioral performance among the aMCI group comparable to HC (see e.g., [73]). These neural findings are consistent with previous studies that demonstrated deficits in alerting and orienting in MCI and/or AD [12], [15], [17], [26]–[35].

Attentional deficits in aMCI were most notable during the flanker conflict component of the ANT-G (i.e., executive control of attention), where both behavioral and neural differences were evident between groups. One must use caution when considering group differences (between patients and healthy controls) when task performance is not equal; differences in neural activity could reflect different approaches and/or strategies to the task [73]. However, the important role of the ACC in conflict resolution (e.g., [74]), as well as the previously observed hypometabolism of the ACC in those who convert from MCI to AD [52]–[55], lend support to our interpretation of the present findings; deficits of executive control of attention may be due to deficits of ACC function. Deficits in the executive control of attention [9], [11], [12], [17], [36]–[42] and executive function, more generally [17], [43]–[49], have previously been documented, though the neural substrates have not been well elucidated. The findings of the present examination suggest that in addition to the other deficits characteristic of aMCI (e.g., [2]), there may be substantial deficits in the executive control network (corresponding to less activation in the medial prefrontal cortex).

Given the behavioral and neural deficits in executive control network observed herein, it is interesting to consider plausible mechanisms for changes to the neural substrates, especially the ACC. Although only a few studies have directly investigated abnormalities in the ACC related to the executive control of attention in AD using functional neuroimaging (e.g., [56]–[58]), there is much indirect evidence that ACC dysfunction underlies the observed behavioral deficits in this population. For example, aberrant activation as well as deactivation of the ACC has been observed when subjects with AD or at risk for AD perform non-attentional tasks that necessitate the involvement of attentional functions [75], [76]. Further, a recent study of grey matter density and white matter integrity found grey matter atrophy in the cingulate cortex, and more interestingly, that deafferentation in the cingulate cortex, along with grey matter integrity in hippocampal and parahippocampal areas is predictive of impairment in cognitive function among patients with AD [59]. A recent longitudinal study has also shown that individuals who convert from MCI to AD show decreased metabolic activity in regions of the ACC [77]. Abnormalities in ACC-related functional networks have also been reported in patients with AD and MCI under various task conditions, though with somewhat inconsistent findings [78]–[83]. Increases in ACC functional connectivity have been attributed to the engagement of alternative networks for task performance (i.e., the plasticity argument [79]), while decreases in connectivity among patients with AD has been explained as a breakdown of the memory [83], default mode [80], [84], and attentional networks [82].

In conjunction with previous findings regarding the potential importance of the ACC in MCI and AD, the present study suggests that behavioral deficits in attentional conflict resolution may be due to hypoactivity during conflict resolution in the medial prefrontal cortex among individuals with aMCI. While there are certainly limitations to the present study, we attempted to simultaneously examine multiple attentional functions while also acquiring fMRI data in a population with aMCI. Given that executive control of attention is critical to determining what information reaches conscious awareness, the present findings might suggest that deficits in the executive control of attention are characteristic of aMCI.

Another important consideration is that of breakdown in the DMN among individuals with aMCI and AD [80], [84], [85]. Prefrontal, lateral temporal, and lateral parietal regions, along with the precuneus show amyloid depositions, altered metabolism, and atrophy in AD progression, as well as having a prominent role in the DMN [85]. Given activation of a task-positive network, in conjunction with deactivation of the DMN, during task demands, some of the present findings might best be explained in the context of greater activation of DMN regions in alerting by the aMCI group and more differentiation between the cue conditions of orienting by the aMCI group. This pattern needs further exploration.

Beyond the ACC and the executive control of attention, our findings suggest more broad deficits of attention. One interesting implication of the current findings, though their preliminary basis cannot be overlooked, is that deficits in memory among those with aMCI and AD [6] may in fact be related to deficits in attention (e.g., [86]). There is an intimate relationship between attention and memory such that the two processes mutually constrain one another [87]. Impaired attentional function, as is evident in early stages of AD [7]–[11], may partially contribute to the notable declines in memory function. If this is accurate, one way to identify those individuals with aMCI who are mostly likely to progress to AD may be to evaluate attentional function using well-validated tasks like the ANT. This hypothesis is supported by the fact that attentional function is predictive of cognitive decline among those in early stages of probable AD [13]. Another potential implication of the relationship between attention and memory among those with aMCI is that attentional and/or cognitive training interventions could potentially delay the conversion to AD (e.g., [88]). Delay in conversion might have profound implications for both the individual and for society, especially given the economic burden of AD [5].

The primary limitation of the current study is the small sample size. Although there were no differences between our larger sample and those for whom we were able to collect fMRI data, one thing to consider is whether the current sample is representative of a particular subclass of individuals with aMCI. Those individuals willing to participate in a research study and undergo an MRI scan may be more functional than their peers who are not so inclined. This may be one reason that our MMSE scores were so similar between the two groups. However, this finding may actually lead to an underestimate of the potential differences between the HC and aMCI groups.

Furthermore some of the neural activation observed may be due to Type I error, even though correction for multiple comparisons was conducted using Monte Carlo simulation methods. Despite these limitations, our observations do suggest some interesting patterns. The neural activity associated with the aMCI minus HC contrast for alerting (e.g., TPJ) and orienting (e.g., posterior parietal regions) is consistent with previous findings showing the involvement of these regions in alerting and orienting [23], despite the absence of behavioral differences. This may suggest impairments and compensatory neural activity in the alerting and orienting networks among individuals with aMCI. Neurobehavioral activity related to alerting and orienting in aMCI necessitates further research. There were both behavioral and corresponding neural deficits in executive control corresponding to the flanker conflict condition of the ANT-G. These findings are consistent with known deficits to the executive control of attention [17] in aMCI and AD. Although preliminary, our findings suggest that deficits in attention, particularly in the executive control network, may have important contributions in the clinical presentation of aMCI and potentially its progression to AD.
